# A guide to selecting high-performing antibodies for CSNK2A1 (UniProt ID: P68400) for use in western blot, immunoprecipitation and immunofluorescence

**DOI:** 10.12688/f1000research.153243.1

**Published:** 2024-07-09

**Authors:** Riham Ayoubi, Maryam Fotouhi, Charles Alende, Vera Ruíz Moleón, Kathleen Southern, Carl Laflamme, NeuroSGC/YCharOS/EDDU collaborative group, ABIF consortium

**Affiliations:** 1 Department of Neurology and Neurosurgery, Structural Genomics Consortium, The Montreal Neurological Institute, McGill University, Montreal, Québec, H3A 2B4, Canada

**Keywords:** UniProt ID P68400, CSNK2A1, Casein kinase II subunit alpha, antibody characterization, antibody validation, western blot, immunoprecipitation, immunofluorescence

## Abstract

Casein kinase II subunit alpha (CSNK2A1), a serine/threonine kinase, phosphorylates multiple protein substrates and is involved in diverse cellular and biological processes. Implicated in various human diseases, high-performing antibodies would help evaluate its potential as a therapeutic target and benefit the scientific community. In this study, we have characterized ten CSNK2A1 commercial antibodies for western blot, immunoprecipitation, and immunofluorescence using a standardized experimental protocol based on comparing read-outs in knockout cell lines and isogenic parental controls. These studies are part of a larger, collaborative initiative seeking to address antibody reproducibility issues by characterizing commercially available antibodies for human proteins and publishing the results openly as a resource for the scientific community. While use of antibodies and protocols vary between laboratories, we encourage readers to use this report as a guide to select the most appropriate antibodies for their specific needs.

## Introduction

Casein kinase II subunit alpha (CSNK2A1), encoded by the *CSNK2A1* gene, is a catalytic subunit of the serine/threonine enzyme, casein kinase 2; essential for cell cycle progression, apoptosis, transcription and viral replication. ^1^^–^^5^ Relevant to the etiology of many diseases, including the identification of two missense mutations in the *CSNK2A1* gene associated with autism spectrum disorder, CSNK2A1 is emerging as a promising biomarker and therapeutic target. ^1^^,^^6^^–^^17^ High-performing antibodies would enable data reproducibility and reliable research findings.

This research is part of a broader collaborative initiative in which academics, funders and commercial antibody manufacturers are working together to address antibody reproducibility issues by characterizing commercial antibodies for human proteins using standardized protocols, and openly sharing the data. ^18^^–^^20^ Here, we evaluated the performance of ten commercially-available antibodies for CSNK2A1 for use in western blot, immunoprecipitation and immunofluorescence, enabling biochemical and cellular assessment of the proteins properties and function. The platform for antibody characterization used to carry out this study was endorsed by a committee of industry and academic representatives. It consists of identifying human cell lines with adequate target protein expression and the development/contribution of equivalent knockout (KO) cell lines, followed by antibody characterization procedures with most of the commercially available antibodies against the corresponding target protein. The standardized consensus antibody characterization protocols are openly available on Protocol Exchange (DOI: 10.21203/rs.3.pex-2607/v1). ^21^

The authors do not engage in result analysis or offer explicit antibody recommendations. A limitation of this study is the use of universal protocols - any conclusions remain relevant within the confines of the experimental setup and cell line used in this study. Our primary aim is to deliver top-tier data to the scientific community, grounded in Open Science principles. This empowers experts to interpret the characterization data independently, enabling them to make informed choices regarding the most suitable antibodies for their specific experimental needs. Guidelines on how to interpret antibody characterization data found in this study are featured on the YCharOS gateway. ^22^

## Results and discussion

Our standard protocol involves comparing readouts from wild-type (WT) and KO cells. ^23^^,^^24^ The first step is to identify a cell line(s) that expresses sufficient levels of CSNK2A1 to generate a measurable signal using antibodies. To this end, we examined the DepMap transcriptomics database to identify all cell lines that express the target at levels greater than 2.5 log _2_ (transcripts per million “TPM” + 1), which we have found to be a suitable cut-off (Cancer Dependency Map Portal, RRID:SCR_017655). The HAP1 cell lines expresses the CSNK2A1 transcript at 7.0 log _2_ (TPM+1) RNA levels, which is above the average range of cancer cells analyzed. Parental and *CSNK2A* KO HAP1 cells were obtained from Horizon Discovery ( Table 1).

**Table 1.** Summary of the cell lines used.

Institution	Catalog number	RRID (Cellosaurus)	Cell line	Genotype
Horizon Discovery	C631	CVCL_Y019	HAP1	WT
Horizon Discovery	HZGHC004051c003	CVCL_SJ92	HAP1	*CSNK2A1*

**Figure 1.**
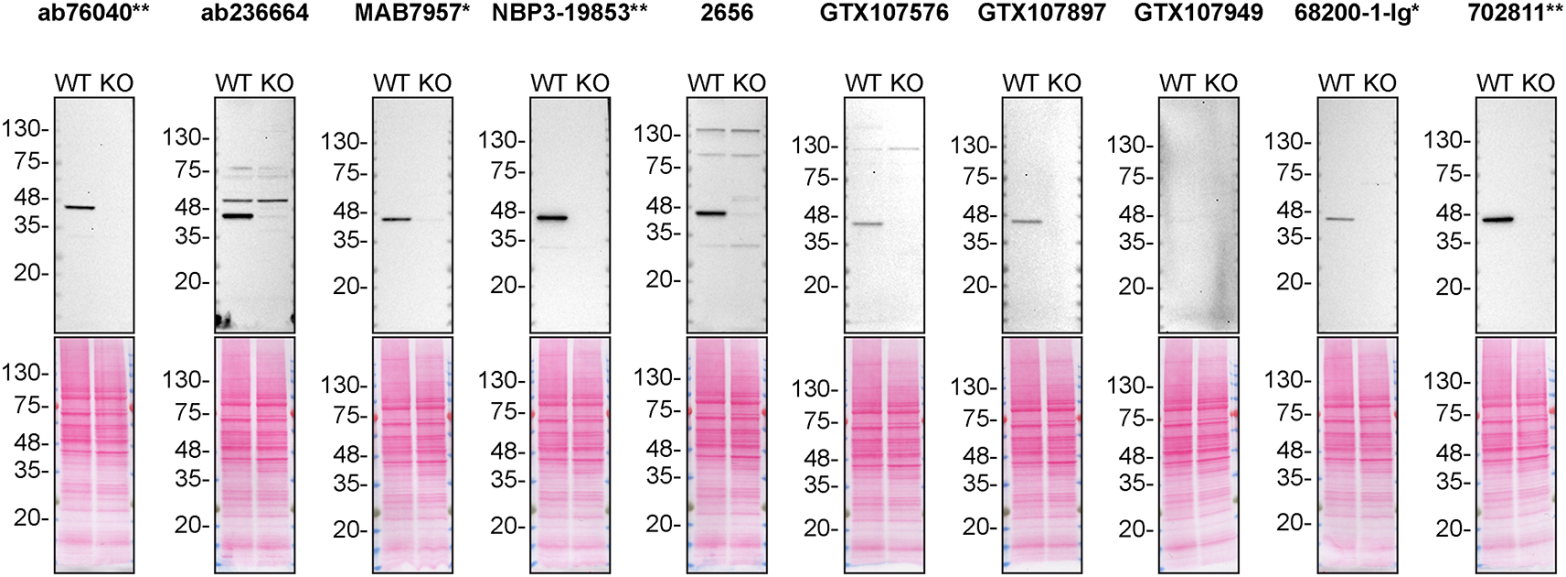
CSNK2A1 antibody screening by western blot. Lysates of HAP1 (WT and *CSNK2A* KO) were prepared and 30 μg of protein were processed for western blot with the indicated CSNK2A1 antibodies. The ponceau stained transfers of each blot are presented to show equal loading of WT and KO lysates and protein transfer efficiency from the polyacrylamide gels to the nitrocellulose membrane. Antibody dilutions were chosen according to the recommendations of the antibody supplier. An exception was given to 68200-1-Ig* recommended at 1/20 000, as the signal was too weak and was therefore diluted and was used at 1/10 000. Antibody dilution used: ab76040** at 1/500, ab236664 at 1/1000, MAB7957* at 1/1000, NBP3-19853** at 1/1000, 2656 at 1/500, GTX107576 at 1/500, GTX107897 at 1/500, GTX107949 at 1/500, 68200-1-Ig* at 1/10 000, 702811** at 1/10 000. Predicted band size: 45 kDa. *Monoclonal antibody, **Recombinant antibody.

For western blot experiments, WT and *CSNK2A* KO protein lysates were ran on SDS-PAGE, transferrred onto nitrocellulose membranes, and then probed with ten CSNK2A1 antibodies in parallel ( Table 2, Figure 1).

**Table 2.** Summary of the CSNK2A1 antibodies tested.

Company	Catalog number	Lot number	RRID (Antibody Registry)	Clonality	Clone ID	Host	Concentration (μg/μl)	Vendors recommended applications
Abcam	ab76040 **	1001668-2	AB_1523361	recombinant-mono	EP1963Y	rabbit	0.16	Wb
Abcam	ab236664	1012742-3	AB_3073947	polyclonal	-	rabbit	2.0	Wb, IP, IF
Bio-Techne	MAB7957 *	CHSN0121081	AB_3073948	monoclonal	844720	mouse	0.5	Wb
Bio-Techne	NBP3-19853 **	230458	AB_3073949	recombinant-mono	S05-7F8	rabbit	0.3	Wb
Cell Signaling Technology	2656	3	AB_2236816	polyclonal	-	rabbit	0.03	Wb
Genetex	GTX107576	40366	AB_10616991	polyclonal	-	rabbit	1.0	Wb
Genetex	GTX107897	40002	AB_1950048	polyclonal	-	rabbit	0.62	Wb, IF
Genetex	GTX107949	39869	AB_2036686	polyclonal	-	rabbit	0.2	Wb
Proteintech	68200-1-Ig *	10028709	AB_2935289	monoclonal	1D5E8	mouse	1.0	Wb
Thermo Fisher Scientific	702811 **	2062784	AB_2734801	recombinant-mono	7H29L3	rabbit	0.5	Wb

Wb = western blot; IF = immunofluorescence; IP = immunoprecipitation.

* Monoclonal antibody.

** Recombinant antibody.

**Figure 2.**
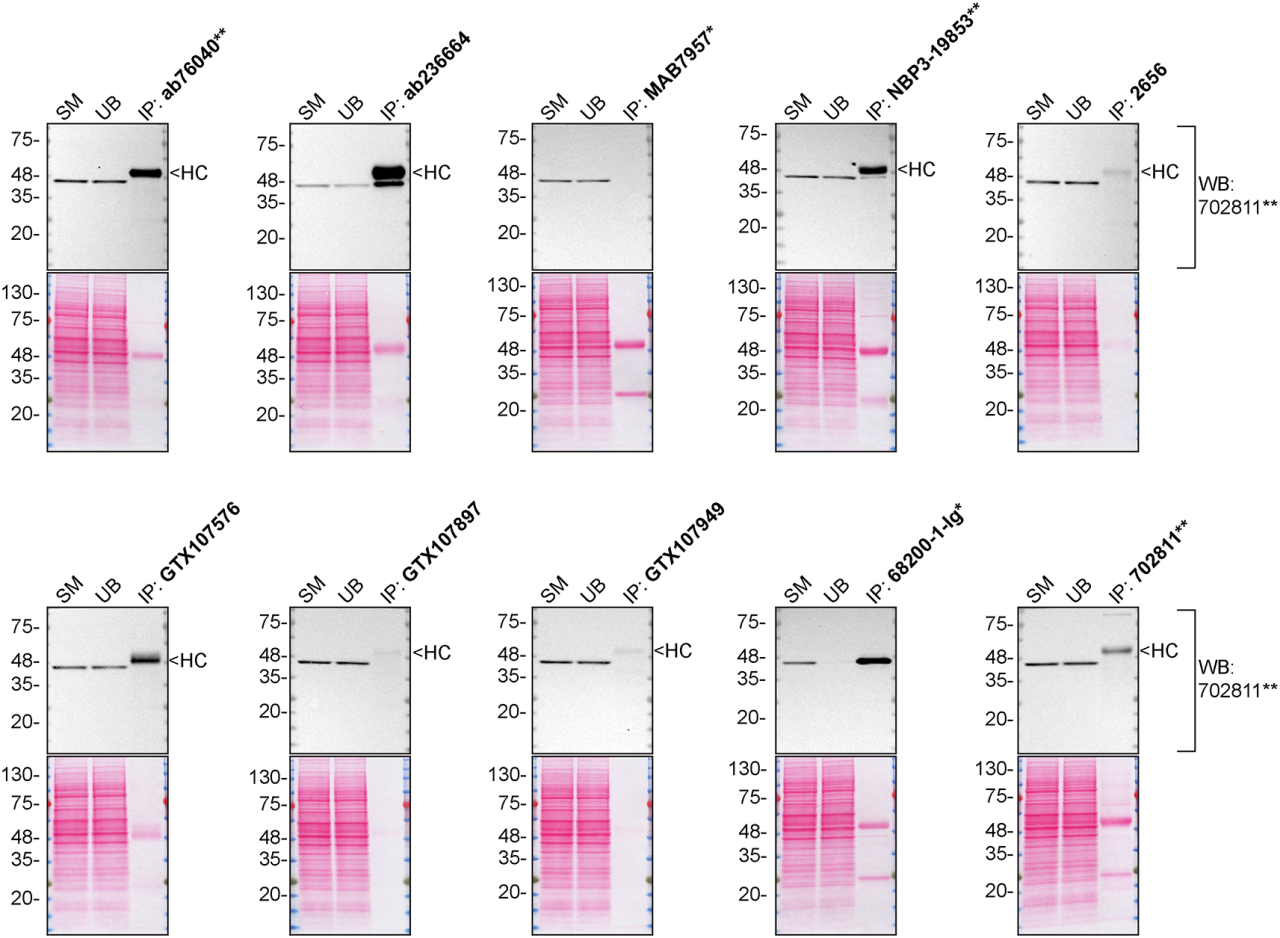
CSNK2A1 antibody screening by immunoprecipitation. HAP1 lysates were prepared, and immunoprecipitation was performed using 2.0 μg of the indicated CSNK2A1 antibodies pre-coupled to Dynabeads protein G or protein A. Samples were washed and processed for western blot with the indicated CSNK2A1 antibody. For western blot, 702811** was used at 1/10 000. The ponceau stained transfers of each blot are shown for similar reasons as in Figure 1. SM = 4% starting material; UB = 4% unbound fraction; IP = immunoprecipitate, HC = antibody heavy chain. *Monoclonal antibody, **Recombinant antibody.

We then assessed the capability of all ten antibodies to capture CSNK2A1 from HAP1 protein extracts using immunoprecipitation techniques, followed by western blot analysis. For the immunoblot step, a specific CSNK2A1 antibody identified previously (refer to Figure 1) was selected. Equal amounts of the starting material (SM), the unbound fraction (UB), as well as the whole immunoprecipitate (IP) eluates were separated by SDS-PAGE ( Figure 2).

**Figure 3.**
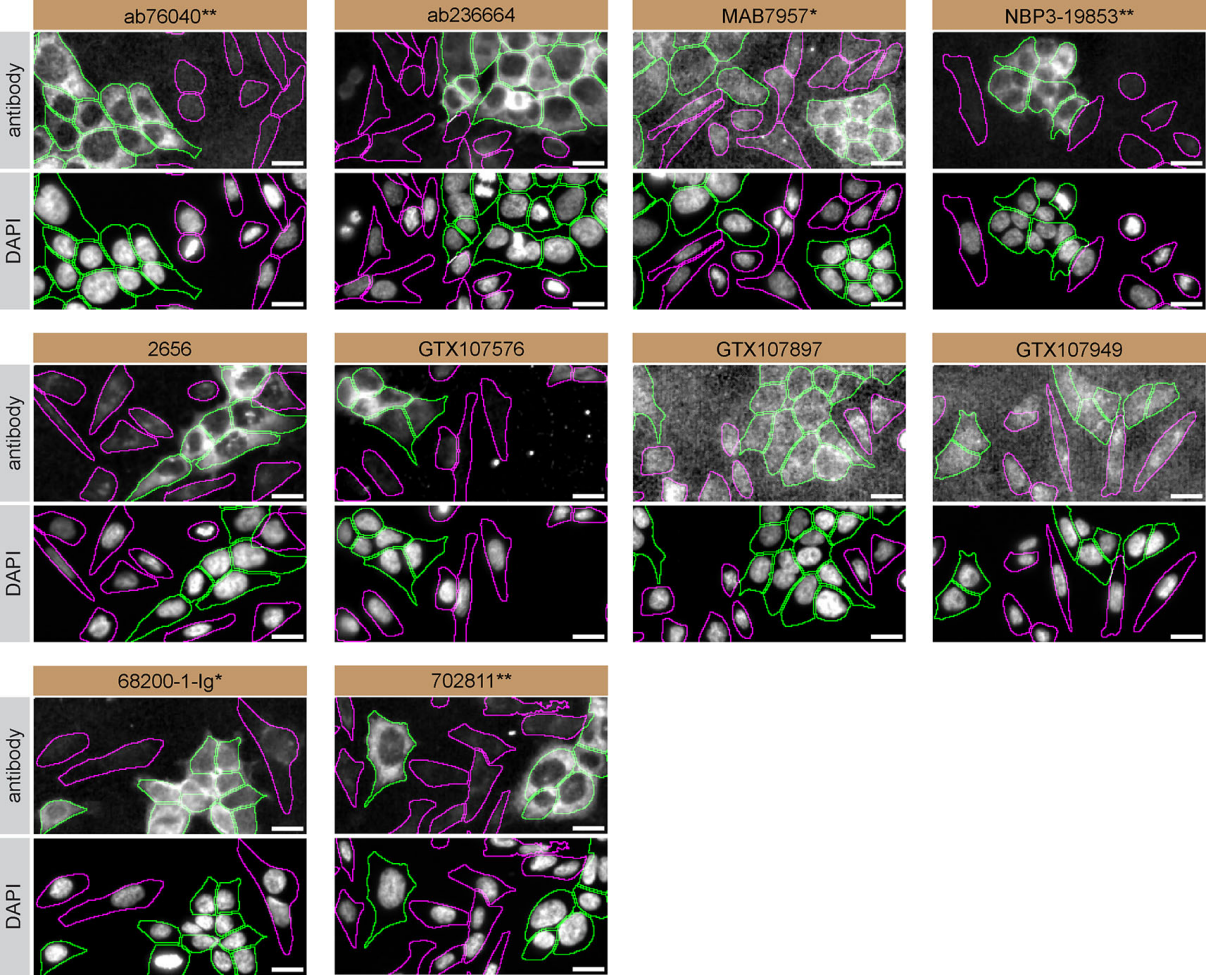
CSNK2A1 antibody screening by immunofluorescence. HAP1 WT and *CSNK2A1* KO cells were labelled with a green or a far-red fluorescent dye, respectively. WT and KO cells were mixed and plated to a 1:1 ratio in a 96-well plate with an optically clear flat-bottom. Cells were stained with the indicated CSNK2A1 antibodies and with the corresponding Alexa-fluor 555 coupled secondary antibody including DAPI. Acquisition of the blue (nucleus-DAPI), green (identification of WT cells), red (antibody staining) and far-red (identification of KO cells) channels was performed. Representative images of the merged blue and red (grayscale) channels are shown. WT and KO cells are outlined with green and magenta dashed line, respectively. When the concentration was not indicated by the supplier, antibodies were tested at concentrations where the signal from each antibody was in the range of detection of the microscope used. Antibody dilution used: ab76040** at 1/500, ab236664 at 1/100, MAB7957* at 1/500, NBP3-19853** at 1/300, 2656 at 1/30, GTX107576 at 1/100, GTX107897 at 1/100, GTX107949 at 1/200, 68200-1-Ig* at 1/500, 702811** at 1/250. Bars = 10 μm. *Monoclonal antibody, **Recombinant antibody.

For immunofluorescence, ten antibodies were screened using a mosaic strategy. First, HAP1 WT and *CSNK2A1* KO cells were labelled with distinct fluorescent dyes in order to distinguish the two cell lines, and the ten CSNK2A1 antibodies were evaluated. Both WT and KO lines were imaged in the same field of view to reduce staining, imaging and image analysis bias ( Figure 3). Quantification of immunofluorescence intensity in hundreds of WT and KO cells was performed for each antibody tested. ^21^ The images presented in Figure 3 are representative of the results of this analysis.

In conclusion, we have screened ten CSNK2A1 commercial antibodies by western blot, immunoprecipitation and immunofluorescence. Several high-quality antibodies that successfully detect CSNK2A1 under our standardized experimental conditions can be identified. Researchers who wish to study CSNK2A1 in a different species are encouraged to select high-quality antibodies, based on the results of this study, and investigate the predicted species reactivity of the manufacturer before extending their research.

The underlying data for this study can be found on Zenodo, an open-access repository for which YCharOS has its own collection of antibody characterization reports. ^27^^,^^28^

## Methods

The standardized protocols used to carry out this KO cell line-based antibody characterization platform was established and approved by a collaborative group of academics, industry researchers and antibody manufacturers. The detailed materials and step-by-step protocols used to characterize antibodies in western blot, immunoprecipitation and immunofluorescence are openly available on Protocol Exchange, a repository dedicated to openly sharing scientific research protocols (DOI: 10.21203/rs.3.pex-2607/v1). ^21^

### Antibodies and cell lines used

Cell lines used and primary antibodies tested in this study are listed in Table 1 and 2, respectively. To ensure that the cell lines and antibodies are cited properly and can be easily identified, we have included their corresponding Research Resource Identifiers, or RRID. ^25^^,^^26^

## Data Availability

Zenodo: Antibody Characterization Report for CSNK2A1, doi.org/10.5281/zenodo.10818214. ^27^ Zenodo: Dataset for the CSNK2A1 antibody screening study, doi.org/10.5281/zenodo.11078556. ^28^ Data are available under the terms of the Creative Commons Attribution 4.0 International license (CC-BY 4.0).
