# Supplementary material for: A guide to selecting high-performing antibodies for CSNK2A1 (UniProt ID: P68400) for use in western blot, immunoprecipitation and immunofluorescence
Source: F1000Res. 2024 Sep 5;13:781. Originally published 2024 Jul 9. [Version 2] doi: 10.12688/f1000research.153243.2 (PMC11472280; doi:10.12688/f1000research.153243.2)
Supplement: Peer review history

# Reviewer report: Reviewer response for version 2

**DOI:** 10.5256/f1000research.170469.r321082

**Published:** 2024-10-10

**Authors:** David Litchfield

**Affiliations:** 1 Western University, London, Ontario, Canada

Based on the revisions that have been made, particularly the inclusion of limitations that explicitly acknowledge the existence of CSNK2A2 (a closely related isoform of CSNK2A1), I am satisfied that the Data Note is now suitable for indexing. Overall, the authors’ systematic evaluation of commercially available antibodies to detect the protein encoded by CSNK2A1 is an important contribution to the field.

Are sufficient details of methods and materials provided to allow replication by others?

Yes

Is the rationale for creating the dataset(s) clearly described?

Yes

Are the datasets clearly presented in a useable and accessible format?

Yes

Are the protocols appropriate and is the work technically sound?

Yes

Reviewer Expertise:

Areas of expertise include signal transduction, protein kinases, kinase inhibitors and phosphoproteomics.  The CK2 family of protein kinases (including CSNK2A1 and CSNK2A2) have represented a central focus of our research.

I confirm that I have read this submission and believe that I have an appropriate level of expertise to confirm that it is of an acceptable scientific standard.

# Reviewer report: Reviewer response for version 1

**DOI:** 10.5256/f1000research.168109.r303889

**Published:** 2024-08-27

**Authors:** Miwako Homma

**Affiliations:** 1 Fukushima Medical University School of Medicine, Fukushima, Japan

This is an interesting study in an area that needs investigating. CK2(CSNK2A1) protein is the first enzyme found to have phosphorylation activity in eukaryotic cells, and it has been shown to participate in various major biological processes. Since there have been several reports of pathologies caused by CSNK2A1 gene mutations, such as Okur-Chung neurodevelopmental syndrome, autism and cancer, high-quality, high-performance antibodies against CSNK2A1 protein are expected to advance research on these diseases. Therefore, the authors conducted the following studies. They characterized ten commercially available antibodies against CSNK2A1 for use: 1. in western blotting under conditions that detect denatured intracellular proteins, 2. in immunoprecipitation to demonstrate antibody recognition of native CSNK2A1 *in vivo*, and 3. in immunofluorescence for cell imaging. However, a few points require clarification and further validation.

1. Antibody evaluations discussed in this manuscript are only comparative observations, and titers of individual antibodies are not quantified. As shown in Figure 1, only one concentration of each antibody diluent was selected, according to the supplier's recommendations. Affinity binding properties are generally verified quantitatively by ELISA, along with Western blotting, in which the intensity of each antibody to the target protein, in this case, purified CSNK2A1, can be calculated by stepwise dilution. I recommend that the authors compare each antibody at the concentration that maximizes performance and that blots be further quantified. It is interesting to note that there are few commercial antibodies that can immunoprecipitate the target molecule, CSNK2A1. However, immunoprecipitation was performed using only one concentration of the indicated antibodies.
2. Although CSNK2A1 (CK2) and CSNK2A2 (CK2’) are encoded at different loci, the amino acid sequences are very similar. Therefore, it is necessary to show that these antibodies are specific for CSNK2A1(CK2) and that they do not also recognize CSNK2A2 (CK2’). For this purpose, I recommend using cells with CSNK2A1 genetically knocked-out (ko) as a control, to show that antibody responses are not observed when only endogenous CANK2A2 is present, or when *CSNK2A2* is expressed as a tagged cDNA in ko cells. Alternatively, antibody performance should be validated using human CSNK2A1 or CSNK2A2 recombinant proteins.

Are sufficient details of methods and materials provided to allow replication by others?

Yes

Is the rationale for creating the dataset(s) clearly described?

Yes

Are the datasets clearly presented in a useable and accessible format?

Yes

Are the protocols appropriate and is the work technically sound?

Yes

Reviewer Expertise:

Intracellular signal transduction, protein kinases, CK2, phospho-proteomics, CK2-ChIP-Seq, RNA-Seq, epigenetics, and prognostic biomarker of cancer.

I confirm that I have read this submission and believe that I have an appropriate level of expertise to confirm that it is of an acceptable scientific standard, however I have significant reservations, as outlined above.

# Reviewer report: Reviewer response for version 1

**DOI:** 10.5256/f1000research.168109.r303880

**Published:** 2024-08-02

**Authors:** David Litchfield

**Affiliations:** 1 Western University, London, Ontario, Canada

This Data Note presents a systematic characterization of a number of commercially available antibodies directed against the gene product of CSNK2A1 (also referred to as the alpha subunit of Casein Kinase II).  As noted by the authors, CSNK2A1 is involved in numerous fundamental biological processes and has been implicated as a promising therapeutic target.  High-quality antibodies against CSNK2A1 and reliable protocols for the use of these antibodies are therefore of considerable importance both for elucidating its biological functions and for advancing efforts to explore the promise of CSNK2A1 as a therapeutic target. Utilization of knockout cell lines to demonstrate that the antibody signal is dependent on the expression of CSNK2A1 provides particularly compelling validation of the specificity of the antibodies.  To further enhance the utility of this report and its accessibility to the research community, I can offer the following suggestions.

  

1. Considering that the gene product encoded by CSNK2A1 is referred to by a variety of names in the published literature in addition to terms adopted by UniProt, it may be beneficial to include additional terms (including CK2alpha and/or CK2α as well as CKIIalpha or CKIIα that are commonly used in the literature). This suggestion is intended to maximize visibility of this article using different search engines.
2. The CSNK2A1 and CSNK2A2 genes encode proteins with very similar enzymatic characteristics and a very high degree of sequence similarity especially within their catalytic kinase domains.  The main differences between the gene products encoded by CSNK2A1 and CSNK2A2 reside within their distinct C-terminal domains that extend beyond their kinase domains.  In reviewing the technical information for some of the antibodies that was available on vendor websites, which in some cases was very limited due to proprietary considerations, it was not entirely clear to me whether some of these antibodies (particularly polyclonal antibodies and/or antibodies raised against recombinant proteins encompassing regions of very high similarity between CSNK2A1 and CSNK2A2) would also detect CSNK2A2 (ie. CK2α’).  Overall, although it does not appear that CSNK2A2 (which represents a protein that is 41 amino acids shorter than the protein encoded by CSNK2A1) is detected by any of the antibodies in Western blots, I believe it would be beneficial to make direct reference to CSNK2A2 in this article and highlight this issue as a potential limitation and/or issue that warrants consideration.  This issue would be particularly pertinent for studies to be performed in the absence of the paired cell lines (i.e., parental and knockout) as  used in this report. 

Minor suggestions.

1. Introduction (line 1) – need to add “kinase” following “serine/threonine” (either to replace “enzyme” or in addition to including “enzyme”)
2. Introduction (line 2).  I would recommend replacing “essential” with less definitive terminology (potentially “with essential roles in”) since CSNK2A1 may not be universally involved in all of the listed processes.   
3. Results and Discussion;  Last sentence of 1 ^st^ paragraph and 1 ^st^ line of 2 ^nd^ paragraph; should be CSNK2A1 (instead of CSNK2A) – especially considering the existence of CSNK2A2 as noted above. 
4. Results and Discussion.  2 ^rd^ paragraph.  “were separated on SDS-PAGE” instead of “ran on SDS-PAGE”
5. Results and Discussion.  3 ^rd^ paragraph.  “equal proportions” rather than “equal amounts”. 

Are sufficient details of methods and materials provided to allow replication by others?

Yes

Is the rationale for creating the dataset(s) clearly described?

Yes

Are the datasets clearly presented in a useable and accessible format?

Yes

Are the protocols appropriate and is the work technically sound?

Yes

Reviewer Expertise:

Areas of expertise include signal transduction, protein kinases, kinase inhibitors and phosphoproteomics.  The CK2 family of protein kinases (including CSNK2A1 and CSNK2A2) have represented a central focus of our research.

I confirm that I have read this submission and believe that I have an appropriate level of expertise to confirm that it is of an acceptable scientific standard, however I have significant reservations, as outlined above.

# Response

**Published:** 2024-08-14

**Authors:** Kathleen Southern

**Affiliations:** McGill University, Canada

Thank you to David Litchfield for reviewing this manuscript. We hope our response to your comments and concerns clarify any misinterpretations and questions you may have previously had. Additionally, the authors have revisited the initial manuscript, including a detailed methods section to ensure the procedure is reproducible. As well, a limitation section has been included to address concerns regarding the inherent limitations of the overall platform as well as when evaluating CSNK2A1 antibodies. A third table has been included to help guide readers interpret the results and select high-performing antibodies, which is the aim of this study. Please refer to version 2.

  

1. *Considering that the gene product encoded by CSNK2A1 is referred to by a variety of names in the published literature in addition to terms adopted by UniProt, it may be beneficial to include additional terms (including CK2alpha and/or CK2α as well as CKIIalpha or CKIIα that are commonly used in the literature). This suggestion is intended to maximize visibility of this article using different search engines.*

Thank you for this suggestion, we are always looking to broaden our audience to increase end-user accessibility of our characterization data to enable researchers to select high-quality antibodies for their research. To this end, we have included the suggested terms to our list of keywords. Please refer to version 2.

*2. The CSNK2A1 and CSNK2A2 genes encode proteins with very similar enzymatic characteristics and a very high degree of sequence similarity especially within their catalytic kinase domains.  The main differences between the gene products encoded by CSNK2A1 and CSNK2A2 reside within their distinct C-terminal domains that extend beyond their kinase domains.  In reviewing the technical information for some of the antibodies that was available on vendor websites, which in some cases was very limited due to proprietary considerations, it was not entirely clear to me whether some of these antibodies (particularly polyclonal antibodies and/or antibodies raised against recombinant proteins encompassing regions of very high similarity between CSNK2A1 and CSNK2A2) would also detect CSNK2A2 (ie. CK2α’).  Overall, although it does not appear that CSNK2A2 (which represents a protein that is 41 amino acids shorter than the protein encoded by CSNK2A1) is detected by any of the antibodies in Western blots, I believe it would be beneficial to make direct reference to CSNK2A2 in this article and highlight this issue as a potential limitation and/or issue that warrants consideration.  This issue would be particularly pertinent for studies to be performed in the absence of the paired cell lines (i.e., parental and knockout) as used in this report.*

 The authors are aware of the potential cross reactivity of the antibodies with CSNK2A2. The advantage of using genetic strategies (WT vs KO) for characterization is that we are able to detect whether the antibodies can or cannot target CSNK2A1 by assessing whether the signal is lost in the KO line. That being said, transparency and reproducibility is at the core of our initiative’s values which is why we have mentioned this factor in the limitations section, included in version 2 of the manuscript.

*Minor suggestions. *

1. *Introduction (line 1) – need to add “kinase” following “serine/threonine” (either to replace “enzyme” or in addition to including “enzyme”)*
2. *Introduction (line 2).  I would recommend replacing “essential” with less definitive terminology (potentially “with essential roles in”) since CSNK2A1 may not be universally involved in all of the listed processes.   *
3. *Results and Discussion;  Last sentence of 1 ^st^ paragraph and 1 ^st^line of 2 ^nd^ paragraph; should be CSNK2A1 (instead of CSNK2A) – especially considering the existence of CSNK2A2 as noted above. *
4. *Results and Discussion.  2 ^rd^paragraph.  “were separated on SDS-PAGE” instead of “ran on SDS-PAGE”*
5. *Results and Discussion.  3 ^rd^paragraph.  “equal proportions” rather than “equal amounts”. *

All of these minor changes have been attended to in version 2 of the manuscript which has been submitted.

# Reviewer report: Reviewer response for version 1

**DOI:** 10.5256/f1000research.168109.r303886

**Published:** 2024-08-01

**Authors:** Odile Filhol, Catherine Pillet

**Affiliations:** 1 Université Grenoble-Alpes, Grenoble-Alpes, France; 2 French Alternative Energies and Atomic Energy Commission, Grenoble, France

The article untitled “A guide to selecting high-performing antibodies for CSNK2A1 for use in western blot, immunoprecipitation and immunofluorescence” describes the characterization of ten CSNK2A1 commercially available antibodies and their indicated protocols.

 As written in the abstract, these studies are part of a larger collaborative initiative that is very interesting, and useful, especially in the case of Casein Kinase 2 for which, some antibodies are sometime not adapted.

 The major concern for me, as a specialist of CK2, is that there is not at least, a comment on the specificity of the antibodies to recognize the CSNK2A2 isoform, that is highly homolog to CSNK2A1. Does the HAP1 cell line used by the authors, express this isoform?

  In the immunoprecipitation assays illustrated in Figure 2, 2 µg of each antibody was used. How did you decide to select 2 µg of antibody to coupled them on beads? However, either in Ponceau red staining or using the same antibody for western blot detection, for some of them, the heavy chain antibody is not visible. This is probably due to the low binding of the antibody. Could you please comment on that in the manuscript?

 In Figure 3, could you show a least one picture representative of the HAP1 WT (green) and CSNK2A1 KO (far red) cells that allow identifying them.

 Minor points:

 In table 2, stars in the second column are not useful since the information is provided in column 5.  Stars and the corresponding legend should be removed.

 In Figure 1 and 3, the antibody dilution should be indicated together with the name of each antibody.

 Except the points raised in the comments above, the results are well presented.

 However, I would have liked to have a useful conclusion, suggesting the best CK2 antibodies in relation to their different applications.

Are sufficient details of methods and materials provided to allow replication by others?

Yes

Is the rationale for creating the dataset(s) clearly described?

Yes

Are the datasets clearly presented in a useable and accessible format?

Partly

Are the protocols appropriate and is the work technically sound?

Yes

Reviewer Expertise:

Cell biology and protein kinase CK2 specialist.

We confirm that we have read this submission and believe that we have an appropriate level of expertise to confirm that it is of an acceptable scientific standard, however we have significant reservations, as outlined above.

# Response

**Published:** 2024-08-14

**Authors:** Kathleen Southern

**Affiliations:** McGill University, Canada

Thank you to Odile Filhol and Catherine Pillet for your thorough analysis of our manuscript presenting antibody characterization data for ten commercial CSNK2A1 antibodies to the scientific community. We hope our response to your comments and concerns clarify any misinterpretations and questions you may have previously had. Additionally, the authors have revisited the initial manuscript and made necessary changes to include a detailed methods section as to ensure the procedure is reproducible. As well, a limitation section has been included to address concerns regarding the nature of the platform and inherent limitations associated with it. Please refer to version 2.

***Major points:***

*The major concern for me, as a specialist of CK2, is that there is not at least, a comment on the specificity of the antibodies to recognize the CSNK2A2 isoform, that is highly homolog to CSNK2A1. Does the HAP1 cell line used by the authors, express this isoform?*

**Author Response:** Based on the characterization table provided by Depmap, HAP1 expresses the CSNK2A2 isoform at 5.8 log _2_(TPM+1).  That being said, this study is shared with the scientific community to help researchers find selective antibodies, already commercially available, for CSNK2A1.  That is why a *CSNK2A1 *KO cell line was selected. To test the ability of the antibodies to recognize the isoform, we would need to test them in a *CSNK2A2* KO line.

*In the immunoprecipitation assays illustrated in Figure 2, 2 µg of each antibody was used. How did you decide to select 2 µg of antibody to coupled them on beads? However, either in Ponceau red staining or using the same antibody for western blot detection, for some of them, the heavy chain antibody is not visible. This is probably due to the low binding of the antibody. Could you please comment on that in the manuscript?*

**Author Response: **The amount of 2 µg is selected based on previous experience with trial and error. 1ug was tested in the past and optimized to 2 µg to limit cross reactivity in the IP lane when performing the western blot for IP.

 As for the ponceau staining, the initial concentrations provided by the manufacturers are followed when adding the 2 µg of antibody. In some cases, adhering to the manufacturers antibody concentrations can result in concentrations that are highly diluted. For example, the 2656 antibody from Cell Signalling Technology sis provided at a concentration of 0.03 µg/ul. Given this low concentration, the amount of antibody is very minimal preventing the heavy chain from being visible.

*In Figure 3, could you show a least one picture representative of the HAP1 WT (green) and CSNK2A1 KO (far red) cells that allow identifying them.*

**Author Response: **All of the underlying data is available in the Dataset, referenced in this manuscript and available on the YCharOS zenodo community. Please refer to the following link: https://doi.org/10.5281/zenodo.11078556 . The addition of Table 3 should also clarify how the WT and KO cells can be identified.

***Minor points:***

*In table 2, stars in the second column are not useful since the information is provided in column 5.  Stars and the corresponding legend should be removed.*

**Author Response: **The stars are used outside throughout the report to highlight the renewable antibodies while also allowing the readers to remember the clonality of each antibody without having to refer back to Table 2.

*In Figure 1 and 3, the antibody dilution should be indicated together with the name of each antibody.*

**Author Response: **The antibody dilutions are indicated with the catalog number of each antibody in the figure legends.

*Except the points raised in the comments above, the results are well presented.*

*However, I would have liked to have a useful conclusion, suggesting the best CK2 antibodies in relation to their different applications.*

**Author Response: **We understand this concern and it is why we have chosen to submit the antibody characterization data in the format of Data Notes. As explained in the F1000 article guidelines, Data Notes do not include result analyses or conclusions. Furthermore, the authors prefer to remain unbiased while being transparent with the results obtained from the experiments. To provide context for readers who do not have experience analyzing results from such characterization methods utilizing knockdown lines as controls, we have included a subsequent Table to demonstrate how one can identify successful vs unsuccessful antibodies in each application. Please refer to Table 3 in the updated submission.
